# Early Progesterone Change Associated With Pregnancy Outcome After Fresh Embryo Transfer in Assisted Reproduction Technology Cycles With Progesterone Level of >1.5 ng/ml on Human Chorionic Gonadotropin Trigger Day

**DOI:** 10.3389/fendo.2020.00653

**Published:** 2020-09-15

**Authors:** Chun-I Lee, Hsiu-Hui Chen, Chun-Chia Huang, Pin-Yao Lin, Tsung-Hsien Lee, Maw-Sheng Lee

**Affiliations:** ^1^Institute of Medicine, Chung Shan Medical University, Taichung, Taiwan; ^2^Division of Infertility, Lee Women's Hospital, Taichung, Taiwan; ^3^Department of Obstetrics and Gynecology, Chung Shan Medical University Hospital, Taichung, Taiwan

**Keywords:** progesterone elevation, fresh embryo transfer, hCG administration, live birth, assisted reproduction technology

## Abstract

Several studies have reported a poor implantation rate for assisted reproduction technology (ART) cycles with elevated progesterone (P4) at the end of the follicular phase. Whether all women with increased P4 on the human chorionic gonadotropin(hCG) trigger day should undergo fresh or frozen embryo transfer (ET) remains to be explored. This study attempted to determine that the P4 level on 2 days before hCG administration and P4 ratio can serve as indicators for fresh ET in normal responders with an elevated P4 level of >1.5 ng/ml on the hCG administration day. This was a retrospective cohort study involving 337 ART cycles with fresh ET for normal responders. Serum P4 levels were measured 2 days prior to hCG day (P4 level I) and on the hCG administration day (P4 level II). The P4 ratio was calculated as follows: P4 ratio = P4 level II / P4 level I. The primary outcome is live birth rate of fresh ET cycles. The ROC curves established that the optimal P4 level I and P4 ratio for pregnancy in ART cycles with high P4 level II were 0.975 ng/ml and 1.62, respectively. Patients with a P4 level I of ≤0.975 ng/ml and P4 ratio of >1.62 were associated with a significantly higher implantation (30.8%, 61/198 vs. 10.3%, 19/184, *p* < 0.001) and live birth rates (51.6%, 33/64 vs. 15.0%, 9/60, *p* < 0.001) compared with those with a P4 level I of >0.975 ng/ml and P4 ratio of ≤1.62. A combination of P4 level I and P4 ratio cutoff values of 0.975 ng/ml and 1.62, respectively, had a positive predictive value (PPV) of 82.5% for pregnancy. In conclusion, fresh ET can be an option for women with an early P4 level I under 0.975 ng/ml and a P4 ratio higher than 1.62, especially for those normal responders with an elevated P4 level II >1.5 ng/ml on the hCG administration day. This approach may shorten the time to pregnancy and reduce the cost of ART cycles.

## Introduction

An elevated serum progesterone (P4) level of >1.5 ng/ml on the day of human chorionic gonadotropin (hCG) administration is related to a significant decrease in the ongoing pregnancy rate following assisted reproduction technology (ART) cycles (1), irrespective of the gonadotropin-releasing hormone (GnRH) agonist or antagonist used in the ART protocol. Several studies indeed have suggested that an elevated P4 level on the hCG administration day is associated with a decreased pregnancy rate (1–7). Nonetheless, others have concluded that elevated P4 is not associated with a reduction of the pregnancy rate (8, 9), especially for hyper-responders (10). The reason for this disagreement may be uncertainty about whether implantation is affected by elevated serum P4 concentrations and uncertainty regarding the discriminative threshold of P4 levels.

The deleterious effect of an elevated P4 level in the late follicular phase is likely to influence endometrial receptivity (11–14), leading to embryo–endometrial asynchrony (15). Although embryo quality is generally not affected by elevated serum P4 (11–14, 16–19), excessively disturbed synchrony between embryo development and endometrial receptivity may reduce implantation and pregnancy rates. Moreover, robust evidence demonstrates that elevated serum P4 induces both advanced endometrial histological maturation (20) and significant endometrial epigenetic expression changes that may affect endometrial receptivity (18, 19, 21) and immune tolerance (22). Altogether, the findings of these studies indicate that elevated P4 levels are associated with negative effects on embryo implantation and pregnancy.

A threshold for an elevated P4 level (1.5 ng/ml or 4.77 nmol/l) was reported by Bosch et al. in 2010 (1). However, questions remain regarding a single P4 threshold for predicting pregnancy success. In cases with a large follicular mass, mean circulating P4 concentrations above the normal range were achieved 2.6 days before hCG administration (23). Even in a normal-responder cycle, in many women, elevated follicular-phase P4 concentrations during ovarian stimulation were equivalent to concentrations observed 2–3 days after a luteinizing hormone (LH) surge (23). In extreme cases, asynchrony between endometrial and embryonic development can be expected, because embryo activation and implantation processes do not start before the luteinization signal (24). Although questions remain regarding the effects of late-follicular-phase P4 elevation, an assessment of changes in the last 48 h of the stimulation cycle may be more informative than a single measurement (25). Therefore, a single P4 threshold of 1.5 ng/ml may not sufficiently explain the effects of elevated P4 on pregnancy rate in all groups. A freeze-all policy or fresh blastocyst transfer (26) may be a solution for women with elevated P4. However, these options mainly depend on the patient's embryo quality and the laboratory equipment for cryopreservation at the ART center. The purpose of this study was to examine other parameters for selecting which patients could undergo fresh embryo transfer (ET) even with elevated P4 on hCG day. Thus, such women could avoid additional protocols or costs and achieve a satisfactory clinical outcome. A trend analysis related to more than one variable of P4 level before hCG day would probably be of greater interest.

To verify the concept of trend analysis for P4 elevation, we examined serum P4 concentrations in normal responders at 48 h before hCG administration and on the hCG administration day and sought to identify the discriminative threshold for pregnancy and implantation.

## Materials and Methods

### Patient Selection

This retrospective, single-center study evaluated the trend and effect of P4 elevation before hCG administration. The study cohort consisted of 710 normal responders receiving ART treatment at Lee Women's Hospital in Taiwan from February 2011 to October 2016. The treatment history and clinical outcomes for all patients were obtained from the database of Lee Women's Hospital before analysis. For analyzing the relationship between P4 level and clinical outcomes after fresh ET, only women with fresh ET were included in the analysis. The inclusion criteria for normal responders were as follows: (1) being women aged 20–38 years, (2) having an antral follicle count (AFC) of more than 5, and (3) having oocyte number of more than 5 and <15. The exclusion criteria were as follows: (1) having ovarian hyperstimulation syndrome (OHSS), (2) experiencing repeated implantation failure, (3) experiencing recurrent miscarriage, and (4) engaging sperm or oocyte donation. The retrospective data analysis was approved by the Institutional Review Board of Chung Shan Medical University, Taichung, Taiwan (CS-17064). ClinicalTrials.gov ID is NCT03317548.

### Controlled Ovarian Stimulation

Controlled ovarian stimulation, oocyte collection, and denudation were performed as described in another study (27). All patients received leuprolide acetate (Lupron; Takeda Chemical Industries, Ltd., Osaka, Japan) 1 mg/day for downregulation starting in the midluteal phase. They subsequently received recombinant follicle stimulating hormone (rFSH, Gonal-F; Serono, Bari, Italy) 225 IU/day from cycle day 3 to day 7 for ovarian stimulation then the dose of rFSH was adjusted according the ovarian response until the injection of 250 μg of hCG (Ovidriell; Serono) 36 h before oocyte retrieval.

### Hormonal Measurements and Ultrasound Scans

On day 3 of the ART treatment cycle, each patient underwent a transvaginal ultrasound evaluation scan and serum determination of follicle-stimulating hormone (FSH), estradiol (E2), P4, and LH. Subsequent follicle development and hormonal determinations were performed on days 6, 8, and 10 of stimulation. Additionally, serum P4 and E2 levels were measured 2 days prior to and on the hCG administration day when three or more follicles reached 17 mm. The level of change (trend analysis) in late-follicular-phase P4 was calculated according to the P4 level measurements 2 days prior to hCG day (P4 level I) and on hCG day (P4 level II).

Hormone assay was performed through a competitive chemiluminescence immunoassay within 2 h of collection by using an immunoassay analyzer (DXI800; Beckman Coulter, CA, USA). The interassay coefficients of variation for FSH, LH, E2 and P4 were 5.1, 5.4, 12, and 7.9%, respectively, whereas the intra-assay coefficients of variation were 3.6, 4.3, 6, and 8.2%, respectively. The functional sensitivity level was 0.2 mIU/ml for FSH, 0.2 mIU/ml for LH, 20 pg/ml for E2, and 0.1 ng/ml for P4.

### Trend Analysis of Progesterone Elevation

P4 level I at 48 h before hCG day and P4 level II on hCG day were measured for analysis in this study. A P4 ratio was then calculated as P4 level II/P4 level I. To analyze the effect of elevated P4 level II on clinical outcomes, the cycles were first divided into two groups according to the serum P4 concentration on hCG day: a group of P4 level II ≤ 1.5 ng/ml and a group of P4 level II >1.5 ng/ml. To further analyze the effect of elevated P4 level I and P4 ratio on clinical outcomes in patients with a high P4 level II after fresh ET, ROC curves were computed to establish the optimal P4 level I and P4 ratio for pregnancy rate in women with elevated P4 level II (*n* = 149). Furthermore, the women with P4 level II > 1.5 ng/ml (*n* = 149) were further divided into subgroups according to P4 level I and P4 ratio cutoff values by using ROC curve analysis, to additionally evaluate the effect of P4 level I and the P4 ratio on clinical outcomes.

### Embryo Culture

Retrieved oocytes were cultured in Quinn's Advantage Fertilization Medium (Sage BioPharma, Inc., Trumbull, CT, USA) with 15% serum protein substitute (SPS, Sage BioPharma) in a triple gas phase of 5% CO_2_, 5% O_2_, and 90% N_2_. Following conventional insemination or intracytoplasmic sperm injection (ICSI), all embryos were further cultured in microdrops of a cleavage medium (Sage BioPharma) with 15% SPS. Patients in this study all underwent a fresh ET protocol. On the morning of day 3 (at 70–72 h after insemination or ICSI), the cleaved embryos were assessed and selected for transfer. The remaining embryos were group cultured in microdrops of a blastocyst medium (Sage BioPharma) with 15% SPS for verification. The embryos were evaluated using scoring systems described in another study (28).

Luteal support was provided by either vaginal P4 (Crinone 8% gel; Serono, Istanbul) twice a day for patients with a E2 higher than 3,600 pg/ml on the day of hCG trigger. Otherwise, the patients received hCG1500 IU on day 3, 6, and 9 after oocyte retrieval plus 100 mg of intramuscular P4 from ET to the pregnancy test. Pregnancy was determined using ß-hCG levels in blood tests performed 15 days after ET, and clinical pregnancy was defined as the presence of a gestational sac with accompanying fetal heartbeat.

### Statistical Analysis

Clinical pregnancies were diagnosed according to the presence of a gestational sac on transvaginal ultrasound scans 5 weeks after oocyte retrieval. A miscarriage between weeks 7 and 20 was defined as an abortion. The implantation rate was defined as the number of fetuses with heart activity after 7 weeks of gestation per transferred embryo. The ability to predict pregnancy rate using P4 level and P4 ratio parameters was assessed using the area under the receiver operator characteristic (ROC_AUC_) curve. Differences between groups regarding age, number of transferred embryos, number of cleaved embryos, and the day 3 good embryo rate were analyzed using Mann–Whitney test and Kruskal-Wallis test. Theχ^2^ test and Fisher's exact test was used to compare the clinical pregnancy, implantation, and live birth rates among groups. The interrelations between the predictor variables were examined using Spearman's rank correlation coefficients. According to the results of correlation coefficients, the variables including the women age, hormone levels, AMH, the MII oocyte number, day 3 embryo quality, total FSH dosage and the number of transferred embryos were included into statistically analysis of logistic regression. The univariate regression analysis was applied to evaluate the effect of a single factor related to the live birth probability. Multivariate relations between predictor variables and the live birth were statistically analyzed in regression models. A *p-*value of <0.05 indicated a significant difference. All calculations were performed using SPSS version 17.0 (StatSoft, Inc., Tulsa, OK, USA).

## Results

### Elevated P4 on hCG Day Was Associated With Decreased Pregnancy and Live Birthrates After Fresh ET

Of the total of 498 normal responders who underwent ART protocols during the study period, 25 patients had no adequate embryos available for ET and 136 patients took freeze all policy due to preimplantation genetic test for aneuploidy (PGT-A). Consequently, 337 fresh ET cycles were recruited for data analysis (Table 1). The difference in female age (33.4 ± 2.8 years vs. 33.1 ± 3.5 years) and ET number (3.0 ± 0.5 vs. 3.1 ± 0.7) between patients with a low P4 (P4 level II of ≤1.5 ng/ml) and those with a high P4 (P4 level II of >1.5 ng/ml) was not significant. Similarly, the difference in day 3 good embryo rate (65.1 ± 22.3 vs. 64.1 ± 26.7%) between the two groups was not statistically significant. The rates of implantation (21.7%, 100/461 vs. 28.8%, 162/562; *p* = 0.009), and live birth (32.2%, 48/149 vs. 44.7%, 84/188; *p* = 0.020) in the high P4 group were significantly lower than those in the low P4 group. The E2 (2,152 ± 999 pg/ml vs. 1,771 ± 793 pg/ml) and LH (2.6 ± 1.9 mIU/ml vs. 2.1 ± 1.3 mIU/ml) levels on hCG administration day in the high P4 group were significantly higher than those in the low P4 group. The average P4 level I in the high P4 level II group (1.23 ± 0.80 ng/ml) was significantly higher than that in the low P4 level II group (0.55 ± 0.22 ng/ml, *p* < 0.001).

**Table 1:** Comparison of hormonal profile and clinical outcomes between the low progesterone (P4) and high P4 groups (P4 levels ≤ 1.5 ng/ml and P4 levels >1.5 ng/ml on the day HCG administration, respectively).

**P4 level II (ng/ml)**	**Low P4 (P4 ≤ 1.5 ng/ml)**	**High P4 (P4 > 1.5 ng/ml)**	***P*-value**
*N* (patients, cycles)	188	149	–
Age (years)	33.4 ± 2.8	33.1 ± 3.5	0.682
**BASAL ENDOCRINE HORMONE LEVELS**			
FSH (mIU/ml)	6.9 ± 2.5	7.5 ± 2.9	0.015
LH (mIU/ml)	4.9 ± 3.3	5.4 ± 3.8	0.258
E2 (pg/ml)	32.4 ± 25.6	29.5 ± 21.5	0.847
P level I	0.55 ± 0.22	1.23 ± 0.80	<0.001
**DAY OF hCG INJECTION**			
LH (mIU/ml)	2.1 ± 1.3	2.6 ± 1.9	0.022
E2 (pg/ml)	1771 ± 793	2152 ± 999	<0.001
P4 (ng/ml) (P level II)	0.68 ± 0.22	2.53 ± 1.25	<0.001
Total FSH dosage	2878 ± 617	2834 ± 753	0.218
**OOCYTE AND EMBRYO QUALITY**			
Oocyte number	9.7 ± 2.7	10.3 ± 3.4	0.113
MII number	8.2 ± 2.5	8.0 ± 3.3	0.379
Total embryo number	6.9 ± 2.5	6.1 ± 2.8	0.004
Day 3 good embryo rate	65.1 ± 22.3	64.1 ± 26.7	0.896
**CLINICAL OUTCOMES**			
ET number	3.0 ± 0.5	3.1 ± 0.7	0.086
Implantation rate	28.8 (162/562)	21.7 (100/461)	0.009
Pregnancy rate	53.7 (101/188)	43.6 (65/149)	0.065
Abortion rate	16.8 (17/101)	28.6 (16/56)	0.082
Live birth rate	44.7 (84/188)	32.2 (48/149)	0.020
Gestational age (weeks)	37.1 ± 2.3	36.9 ± 2.2	0.430
Birth weight (g)	2,608 ± 582	2,681 ± 652	0.663

*Statistical methods: t-test, χ^2^ test and Fisher's exact test*.

*In the table, percentages are used to describe categorical data and mean ± SD used to describe a set of continuous data*.

*P level II, the progesterone levels on the hCG day; FSH, follicle stimulating hormone; E2, estrogen; LH, luteinizing hormone; P4, progesterone; ET, embryo transfer. MII, oocyte with metaphase II*.

### Early Serum P4 Level I and P4 Ratio Were Better Predictors of Pregnancy and Live Birth Than P4 Level II

**Figure 1:**
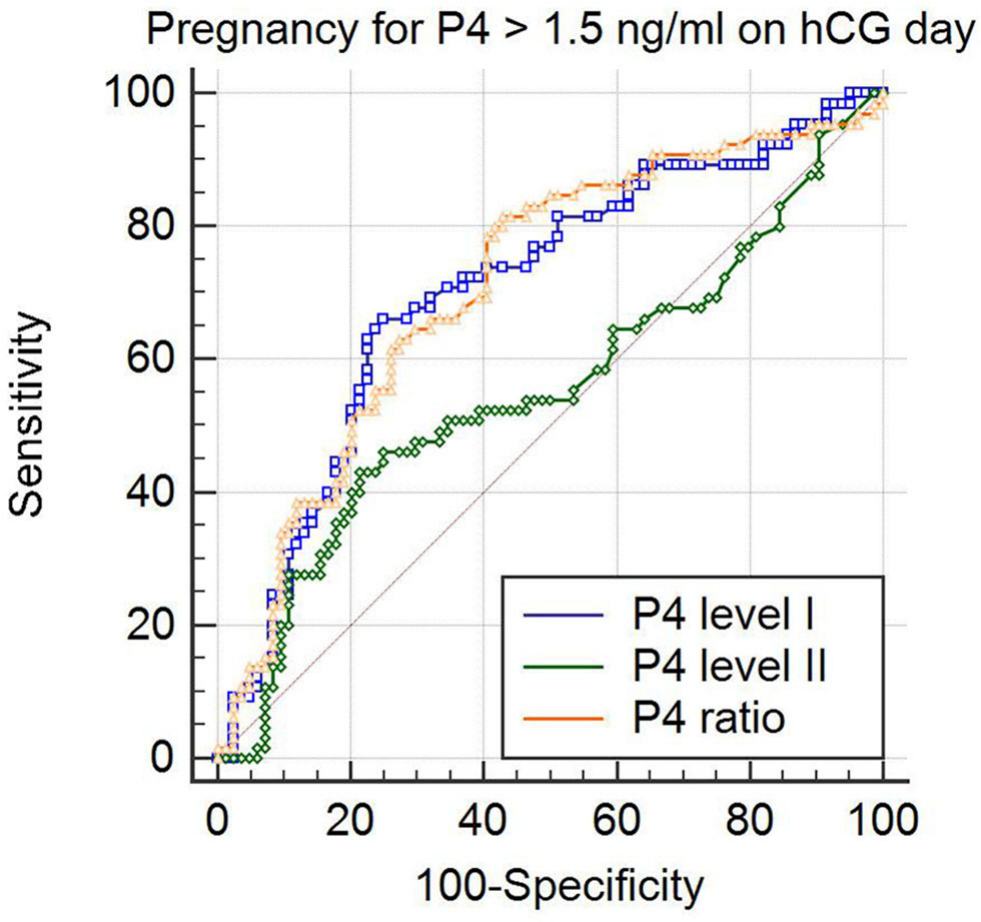
Comparison of predictive values for pregnancy by analysis of the area under receiver operator characteristic (ROC) curve (ROC_AUC_) in the patients with progesterone levels > 1.5 ng/ml on the day of hCG injection. The ROC_AUC_ of the progesterone on 2 days before hCG trigger (P4 level I) and that of the P4 ratio (P4 level II/ P4 level I) was significantly larger than that of progesterone on the day of hCG (P4 level I vs. P4 level II; *p* = 0.02 and P4 ratio vs. P4 level II; *p* < 0.001).

To analyze the clinical outcomes in women with elevated P4 level II (>1.5 ng/ml; *n* = 149) after fresh ET, ROC curves were used to establish the optimal P4 level I and P4 ratio for pregnancy rate in such women. The ROC curve established that the optimal P4 level I for pregnancy rate was under 0.975 ng/ml, with an area under the curve (AUC) of 0.705 (95% confidence interval (CI): 0.625–0.777; Table 2 and Figure 1), sensitivity of 66.1%, specificity of 74.7%, positive predictive value (PPV) of 65.1%, and negative predictive value (NPV) of 75.6%. For the P4 ratio, the ROC curve revealed an AUC of 0.712 (95% CI: 0.632–0.783), sensitivity of 81.5%, specificity of 57.1%, PPV of 79.4%, and NPV of 59.3%. Moreover, the AUC for P4 level II was 0.554 and thus significantly lower than those for P4 level I and the P4 ratio (*p* = 0.02 and *p* < 0.001, respectively). A combination of P4 level I (0.975 ng/ml) and P4 ratio (1.62) was predictive of pregnancy, with a PPV of 82.5%.

**Table 2:** Diagnostic efficacy for progesterone (P4) ratio, P4 level I, and P4 level II for the pregnancy outcome in the high P4 group (*n* = 149) by comparison of AUC^ROC^ (the area under the ROC curve).

	**P4 level I**	**P4 level II**	**P4 ratio**
Sensitivity (%)	66.1	43.1	81.5
Specificity (%)	74.7	78.6	57.1
AUC^ROC^	0.705 ^a^	0.554 ^a^^,^^b^	0.712 ^b^
95% CI	0.625–0.777	0.470–0.635	0.632–0.783
Cut-off value	0.975	2.680	1.62
PPV (%)	65.1	36.9	79.4
NPV (%)	75.6	81.0	59.3
LR+	2.62	2.01	1.90
LR-	0.45	0.72	0.32

*Statistical methods: z-test*.

*Same superscript in the same row indicates statistical significance*,

a *p = 0.02*,

b *p < 0.001*.

*P4 level I, the progesterone levels on 2 days prior to hCG day; P4 level II, progesterone level on the day of hCG administration; AUC^ROC^, the area under the ROC curve; ROC, receiver operating characteristic; CI, Confidence Interval; PPV, positive predicative value; NPV, negative predicative value; LR+, Likelihood ratio for positive test results; LR-, Likelihood ratio for negative test results*.

According to ROC curve analysis, the optimal P4 level I and P4 ratio cutoff values for women with elevated P4 level II were 0.975 ng/ml and 1.62, respectively. Women with P4 level I ≤ 0.975 ng/ml had significantly higher rates of implantation (30.8%, 61/198 vs. 14.8%, 39/263; *p* < 0.001), pregnancy (67.2%, 43/64 vs. 25.9%, 22/85; *p* < 0.001), and live birth (51.6%, 33/64 vs. 17.6%, 15/85; *p* < 0.001; Table 3) than women with P4 level I >0.975 ng/ml did. Similarly, women with a P4 ratio of >1.62 had significantly higher rates of implantation (29.2%, 81/277 vs. 10.3%, 19/184; *p* < 0.001), pregnancy (59.6%, 53/89 vs. 20.0%, 12/60; *p* < 0.001), and live birth (43.8%, 39/89 vs. 15.0%, 9/60; *p* < 0.001; Table 4) than women with a P4 ratio of ≤1.62 did.

**Table 3:** Comparison of the clinical outcome between progesterone (P4) level I ≤ 0.975 ng/ml and > 0.975 ng/ml groups for the high P4 group (*n* = 149).

**P4 level I (ng/ml)**	**≤0.975**	**>0.975**	***P*-value**
N (patients, cycles)	64	85	–
Age (years)	33.2 ± 3.4	33.0 ± 3.5	0.571
P4 level II (ng/ml, hCG day)	2.78 ± 1.32	2.34 ± 1.15	0.259
P4 ratio	6.17 ± 5.74	1.53 ± 0.77	<0.001
LH (mIU/ml, hCG day)	2.6 ± 1.8	2.5 ± 2.0	0.302
E2 (pg/ml, hCG day)	1944 ± 1004	2308 ± 972	0.021
Total FSH dosage	2802 ± 912	2859 ± 611	0.118
Oocyte number	9.5 ± 3.6	10.9 ± 3.3	0.018
MII no.	7.5 ± 3.5	8.3 ± 3.1	0.111
Total embryo no.	6.0 ± 3.0	6.2 ± 2.7	0.398
Day 3 good embryo rate	64.8 ± 27.0	63.5 ± 26.6	0.758
ET no.	3.1 ± 0.7	3.1 ± 0.6	0.956
Implantation rate	30.8 (61/198)	14.8 (39/263)	<0.001
Pregnancy rate	67.2 (43/64)	25.9 (22/85)	<0.001
Abortion rate	23.3 (10/43)	27.3 (6/22)	0.723
Live birth rate	51.6 (33/64)	17.6 (15/85)	<0.001
Gestational age (weeks)	37.0 ± 2.3	36.7 ± 2.0	0.506
Birth weight (g)	2,732 ± 684	2,570 ± 582	0.430

*Statistical methods: Mann-Whitney test and χ^2^test*.

*In the table, percentages are used to describe categorical data and mean ± SD used to describe a set of continuous data*.

*P4 level I, the progesterone levels on 2 days prior to hCG day; P4 level II, progesterone level on the day of hCG administration; FSH, follicle stimulating hormone; E2, estrogen; LH, luteinizing hormone; P4, progesterone; ET, embryo transfer; MII, oocyte with metaphase II*.

**Table 4:** Comparison of the clinical outcome for the patients with high P4 levels on the day of hCG injection (*n* = 149) between P4 ratio ≤1.62 and >1.62 groups.

**P4 ratio**	**≤1.62**	**>1.62**	***P*-value**
N (patients, cycles)	60	89	–
Age (years)	33.1 ± 3.6	33.1 ± 3.4	0.898
P4 level I (ng/ml)	1.82 ± 0.84	0.82 ± 0.43	<0.001
P4 level II (ng/ml, hCG day)	2.02 ± 0.88	2.87 ± 1.34	<0.001
LH (mIU/ml, hCG day)	2.3 ± 1.6	2.8 ± 2.1	0.165
E2 (pg/ml, hCG day)	2,441 ± 905	1,957 ± 1,017	0.004
Total FSH dosage	2,880 ± 565	2,804 ± 858	0.176
Oocyte number	11.0 ± 3.2	9.8 ± 3.6	0.051
MII no.	8.3 ± 3.2	7.7 ± 3.4	0.174
Total embryo no.	6.3 ± 2.8	6.0 ± 2.8	0.352
Day 3 good embryo rate	62.2 ± 25.0	65.3 ± 27.8	0.434
ET no.	3.1 ± 0.6	3.1 ± 0.7	0.645
Implantation rate	10.3 (19/184)	29.2 (81/277)	<0.001
Pregnancy rate	20.0 (12/60)	59.6 (53/89)	<0.001
Abortion rate	16.7 (2/12)	26.4 (14/53)	0.714
Live birth rate	15.0 (9/60)	43.8 (39/89)	<0.001
Gestational age (weeks)	37.7 ± 1.5	36.7 ± 2.3	0.255
Birth weight (g)	2,821 ± 477	2,648 ± 687	0.452

*Statistical methods: Mann-Whitney test, χ^2^ test and Fisher's exact test*.

*In the table, percentages are used to describe categorical data and mean ± SD used to describe a set of continuous data*.

*P4 level I, the progesterone levels on 2 days prior to hCG day; P4 level II, progesterone level on the day of hCG administration; FSH, follicle stimulating hormone; E2, estrogen; LH, luteinizing hormone; P4, progesterone; ET, embryo transfer; MII, oocyte with metaphase II*.

After ROC analysis to establish the optimal P4 level I and P4 ratio cutoff values, we noted that there was no P4 level I higher than 0.975 in the P4 level II ≤ 1.5 ng/ml group (range: 0.09–0.95 ng/ml; Table 2).

### P4 Ratio ≤1.62 Combined With P4 Level I > 0.975 ng/ml Had the Poorest Clinical Outcomes After Fresh Day 3 ET

When combining two factors, namelyP4 level I and the P4 ratio, to assess their effect on clinical outcomes (Table 5), we subdivided the patients into three groups: those with P4 level I ≤ 0.975 ng/ml and P4 ratio > 1.62 (Group A; *n* = 64 cycles); those with P4 level I > 0.975 ng/ml and P4 ratio ≤ 1.62 (Group B; *n* = 60 cycles); and those with P4 level I > 0.975 ng/ml and P4 ratio > 1.62 (Group C; *n* = 25 cycles). Implantation (30.8%, 61/198 vs. 10.3%, 19/184, *p* < 0.001), pregnancy (67.2%, 43/64 vs. 20.0%, 12/60, *p* < 0.001) and live birth rates (51.6%, 33/64 vs. 15.0%, 9/60, *p* < 0.001) in Group A were significantly higher than those in Group B. Although no significant difference in implantation rates (30.8%, 61/198 vs. 25.3%, 20/79, *p* > 0.05) was observed between Groups A and C, the pregnancy (67.2%, 43/64 vs. 40.0%, 10/25, *p* = 0.030), and live birth rates (51.6%, 33/64 vs. 24.0%, 6/25, *p* = 0.031) in Group A were significantly higher than those in Group C. The implantation rate (25.3%, 20/79 vs. 10.3%, 19/184, *p* = 0.002) in group C was significantly higher than that in Group B, but the difference in pregnancy and live birthrates between these two groups was not significant.

**Table 5:** The effects of combined two factors (P4 ratio and P4 level I) on the outcomes of assisted reproduction technology cycles for the patients with high P4 levels on the day of hCG injection (*n* = 149).

**P4 level I**	**≤0.975**	**>0.975**	
**P4 ratio**	**>1.62**	**≤1.62**	**>1.62**
**Group**	**A**	**B**	**C**
N (patients, cycles)	64	60	25
Age (years)	33.3 ± 3.4	33.1 ± 3.6	32.8 ± 3.5
P4 level II (ng/ml, hCG day)	2.78 ± 1.32^a^	2.02 ± 0.88^a^^,^^b^	3.10 ± 1.39^b^
P4 ratio	6.2 ± 5.8^c^^,^^d^	1.2 ± 0.3^c^^,^^e^	2.3 ± 1.0^d^^,^^e^
LH (mIU/ml, hCG day)	2.6 ± 1.8	2.3 ± 1.6	3.1 ± 1.4
Estradiol (pg/ml, hCG day)	1,944 ± 1,004^f^	2,441 ± 905^f^	1,989 ± 1,070
Total FSH dosage	2,802 ± 912	2,880 ± 565	2,808 ± 720
Oocyte number	9.5 ± 3.6	11.0 ± 3.2	10.6 ± 3.5
Mature oocyte no.	7.5 ± 3.5	8.3 ± 3.2	8.1 ± 3.1
Total embryo no.	6.0 ± 3.0	6.3 ± 2.8	6.0 ± 2.4
Day 3 good embryo rate	64.8 ± 27.0	62.2 ± 25.0	66.6 ± 30.5
ET no.	3.1 ± 0.7	3.1 ± 0.6	3.2 ± 0.7
Implantation rate	30.8 (61/198)^g^	10.3 (19/184)^g^^,^^h^	25.3 (20/79)^h^
Pregnancy rate	67.2 (43/64)^i^^,^^j^	20.0 (12/60)^i^	40.0 (10/25)^j^
Abortion rate	23.3 (10/43)	16.7 (2/12)	40.0 (4/10)
Live birth rate	51.6 (33/64)^k^^,^^l^	15.0 (9/60)^k^	24.0 (6/25)^l^
Gestational age (weeks)	37.0 ± 2.3	37.7 ± 1.5^l^	35.2 ± 1.8^k^^,^^l^
Birth weight (g)	2,732 ± 684	2,821 ± 477	2,194 ± 551

Statistical methods: Kruskal-Wallis test, χ^2^ test and Fisher's exact test (

a *p = 0.026*;

d *p = 0.026*;

f *p = 0.014*;

h *p = 0.002*;

j *p = 0.030*;

b,c,e,g,i,k *p < 0.001*;

l *p = 0.031)*.

*In the table, percentages are used to describe categorical data and mean ± SD used to describe a set of continuous data*.

*P4 level I: 2 days before the day of hCG administration; P4 level II: progesterone level on the day of hCG administration; FSH, follicle stimulating hormone; E2, estrogen; LH, luteinizing hormone; P4, progesterone; ET, embryo transfer; MII, oocyte with metaphase II*.

### Decision Tree Models and Logistic Regression Models

**Figure 2:**
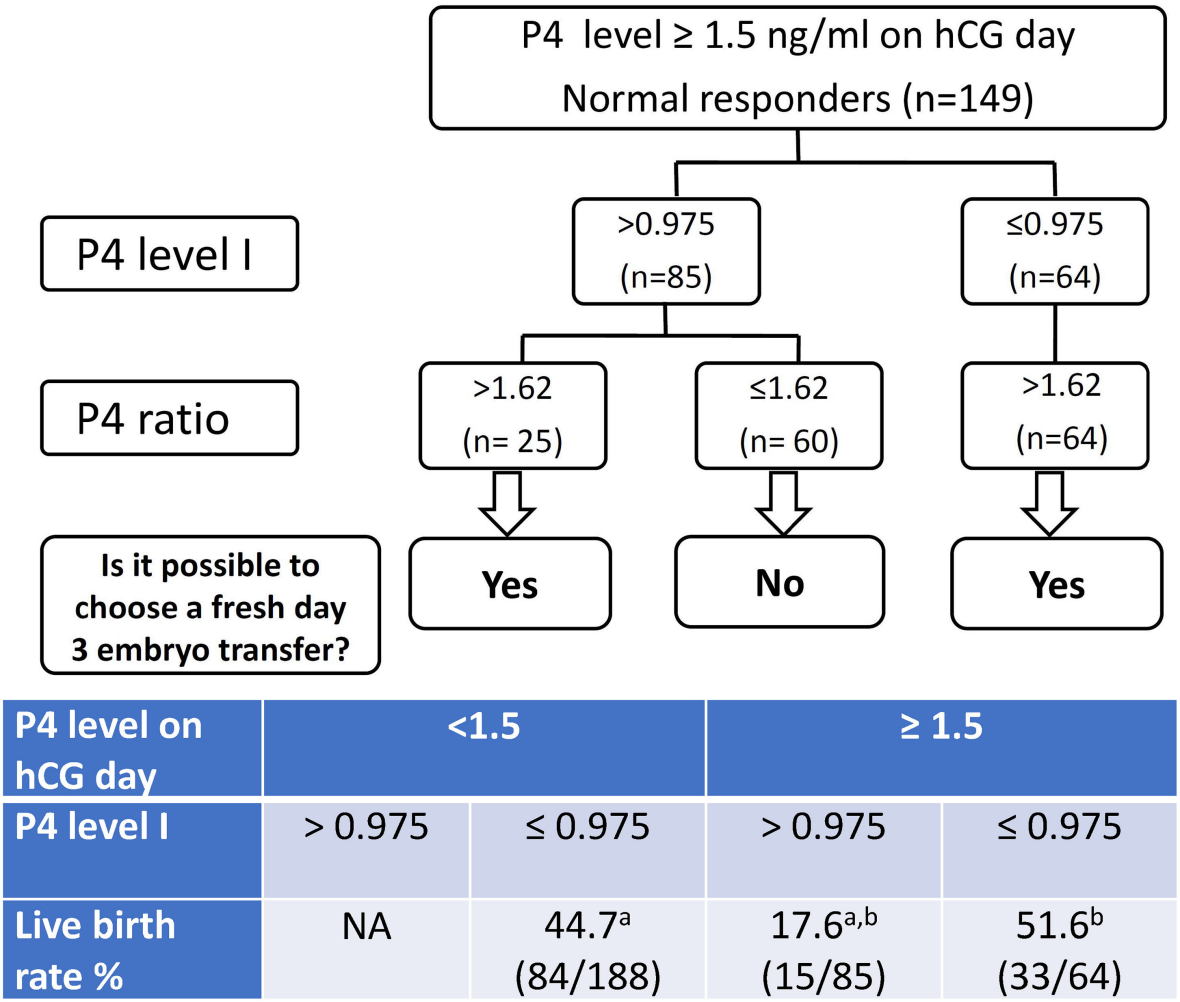
The decision tree models for freeze all or fresh embryo transfer (ET) patients with progesterone levels > 1.5 ng/ml on the day of hCG injection. P4 level I denote the progesterone (P4) level 2 days before the day of hCG administration. P4 level II denote the P4 level on the day of hCG injection. P4 ratio denote P4 level II/ P4 level I. Corresponding live birth rates are listed in the table. ^a,b^*p* < 0.001 by χ^2^ test.

According to the data in the present study, we recommend the decision tree models demonstrated in Figure 2 for the patients with a high P4 on the day of hCG administration. The overall live birth rates (39.2%, 132/337) of the studied group was regarded as the population reference. If we made the decision of freeze all for the high P4 group (P4 > 1.5 ng/mL on hCG day), we would have 188 fresh ET and 84 live birth in this cohort of 337 patients after the first ART cycles. In other words, we would obtain an overall live birth rate 24.9% (84/337) per initiated cycle and a live birth rate 44.7% (84/188) per fresh ET.

If we used the P4 level I for further decision-making process, we would have 252 fresh ET and 117 live birth in this cohort. That means an overall live birth rate 34.7% (117/337) per initiated cycle and a live birth rate 46.4% (117/252) per fresh ET. If we also took the P4 ratio into the decision model, we would have 277 fresh ET and 123 live birth in this cohort of 337 patients after the first ART cycles, which featured an overall live birth rate 36.5% (123/337) per initiated cycle and a live birth rate 44.4% (123/277) per fresh ET.

Consequently, the live birth rate 24.9% per initiated cycle determined by a single high P4 level II in the decision model is significantly lower compared to the 39.2% live birth rate of the overall studied cohort. When the P4 level I or (P4 level I plus P4 ratio) were taken into consideration, the live birth rates per initiated cycle (34.7 and 36.5%, respectively) are not significantly different from the 39.2% of the overall population.

According to univariate logistic regression analysis, the P4 level I (OR: 0.383, 95% CI: 0.2342–0.629, *p* < 0.001; Table 6) was negatively associated with livebirth probability; but the number of day 3 good embryo (OR: 1.150, 95% CI: 1.040–1.272, *p* = 0.006), was positively associated with the livebirth probability. According to multivariate logistic regression analysis of several key factors, only the P4 level I (OR: 0.315, 95% CI: 0.178–0.556, *p* < 0.001) and the number of day 3 good embryo (OR: 1.135, 95% CI: 1.000–1.287, *p* < 0.049) were the significant predictor in live birth probability.

**Table 6:** Univariate and Multivariate regression analysis of factors related to the live birth probability.

**Variables**	**B**	**Odds ratio**	**95% CI**	***p*-value**
**Univariate regression analysis**				
Women age (year)	−0.038	0.963	0.897–1.033	0.290
Duration (year)	−0.070	0.933	0.852–1.021	0.129
FSH (mIU/ml) (Basal level)	0.027	1.028	0.948–1.115	0.506
LH (mIU/ml) (Basal level)	−0.010	0.990	0.930–1.054	0.763
E2 (pg/ml) (Basal level)	−0.010	1.013	0.880–1.167	0.854
AMH (ng/ml)	0.013	0.990	0.980–1.000	0.061
P level I (ng/ml)	−0.959	0.383	0.234–0.629	<0.001
LH (hCG day)	0.024	1.024	0.894–1.173	0.733
E2 (hCG day)	<0.001	1.000	1.000–1.000	0.807
P level II (hCG day)	−0.116	0.891	0.742–1.069	0.214
P ratio	0.052	1.053	0.981–1.131	0.153
MII number	0.064	1.066	0.987–1.151	0.105
D3GE number	0.140	1.150	1.040–1.272	0.006
FSH dosage (IU)	0.000	1.000	1.000–1.000	0.361
Number of transferred embryos	0.159	1.173	0.804–1.711	0.409
**Variables**	**B**	**Odds ratio**	**95% CI**	***p*****–value**
**Multivariate regression analysis**				
Women age (year)	−0.067	0.935	0.805–1.011	0.091
P level I	−1.156	0.315	0.178–0.556	<0.001
P level II	0.081	1.084	0.875–1.343	0.461
MII number	0.031	1.032	0.934–1.140	0.539
D3GE number	0.126	1.135	1.000–1.287	0.049
Number of transferred embryos	0.190	1.210	0.781–1.873	0.393

## Discussion

For normal responders after fresh ET, an elevated P4 level II (>1.5 ng/ml) appears to be correlated with reduced pregnancy and live birth rates. However, not all patients with elevated P4 level II exhibit poor clinical outcomes. In the present study, the group of women with P4 level I ≤ 0.975 ng/ml and P4 ratio > 1.62 demonstrated the best pregnancy and delivery rates after fresh ET, even though they had an elevated P4 level II on hCG day. Assessment of the P4 level change during the last 48 h of the stimulation cycle was more informative than a single P4 level measurement on hCG day. Therefore, we suggest thatP4 level I (cutoff value: 0.975 ng/ml) on 2 days before hCG administration and an elevated P4 ratio (cutoff value for P4 ratio: 1.62) may be appropriate diagnostic tools for predicting implantation and live birth rates even in normal responders with an elevated P4 level II on hCG trigger day.

The low reliability of the P4 level on hCG day rendered it a poor predictor of clinical outcomes (29) because only one cutoff value was considered. When comparing P4 level I between the P4 level II > 1.5 ng/ml and ≤ 1.5 ng/ml groups, we found that the mean P4 level I in women with high P4 levels was significantly higher than that in women with low P4 levels (Table 1). In addition, ROC curve analysis in this study indicated that a single P4 level II measured on hCG day was unable to predict pregnancy in fresh ET cycles (AUC = 0.554). By contrast, P4 level I showed a better predictive capability for pregnancy after fresh ET in normal responders with elevated P4 level II. Moreover, according to the cutoff value for P4 level I, we found that the group of patients with a low P4 level II (≤1.5 ng/ml) all demonstrated a low P4 level I (≤0.975 ng/ml) as well. These results indicate that the cutoff value for P4 level I may be a predictor of not only pregnancy outcomes after fresh ET but also P4 level II elevation.

A high serum P4 level induces both advanced endometrial maturation (20) and gene expression (21, 30) and produces asynchrony between endometrial maturation and the embryo, which may result in reduced implantation (31). Although the detrimental effects of elevated P4 on endometrium and pregnancy are well-known, its effects starting before or at hCG day have been less frequently explored. One study reported that premature luteinization based on elevated serum P4 on hCG day is a common occurrence reflecting healthy follicular development and is associated with increased pregnancy rates in oocyte donors (32). Furthermore, even women with a normal follicular mass and FSH exhibited mean circulating P4 concentrations that exceeded the normal range 0.6 and 0.9 days before hCG administration, respectively (23). According to the results of this study, we suggest that P4 level I on 2 days before hCG administration may be an early signal, which reflects its detrimental effects on endometrium maturation and early luteinization and for the assessment of pregnancy rate which impaired by P4 elevation.

We also observed that a P4 ratio of >1.62 indicates that a group exhibiting elevated P4 on 2 days before hCG day has significantly higher implantation, pregnancy, and live birth rates than a group with a low P4 ratio (≤1.62) does. The P4 ratio represents the change in P4 level during the 48 h before hCG day, and a P4 level elevation of more than 1.62-fold would engender better pregnancy and live birth rates. Demir et al. (25) observed more stable P4 values in pregnancy cycles during the last 48 h of stimulation, which differed from the fluctuations observed in non-pregnancy cycles. They suggested such fluctuations could be another factor affecting pregnancy outcomes. Embryo–endometrium interaction typically undergoes certain hormone-dependent changes during the implantation window. P4 transforms endometrial epithelia into secretory tissue (33, 34) and triggers the expression of a unique set of genes during implantation and pregnancy. Therefore, we suggest that the P4 ratio pattern indicates a P4 level sufficient to regulate genes associated with implantation and that increasing P4 ratio change may be related to luteal phase support for maintaining pregnancy. However, one essential rule is that P4 level I have not elevated which may impair endometrial receptivity early. This could explain why the implantation rates in Groups A (P4 level I ≤ 0.957, P4 ratio > 1.62) and C (P4 level I > 0.975 ng/ml, P4 ratio > 1.62) did not significantly differ, whereas the live birth rate in group C was significantly lower than that in Group A. Accordingly, monitoring serum P4 levels in the late follicular phase of ART would seem to be highly beneficial.

Moreover, according to our results, P4 level I demonstrated a better NPV (75.6%) than P4 ratio (Table 2), whereas P4 ratio had a better PPV (79.4%) for women with P4 level II > 1.5 g/ml. All women with P4 level I ≤ 0.975 ng/ml exhibited a P4 ratio of > 1.62, even those with elevated P4 level II. These results indicate that P4 level I may serve as a selection tool for fresh ET prior to oocyte retrieval. Nonetheless, we maintain that the P4 ratio is also necessary in selecting patients for fresh ET because it demonstrates satisfactory PPV and that combining the two factors could predict the group with the poorest clinical outcome after fresh ET. Along with the threshold value of P4 level I, the P4 ratio can serve as a predictor of pregnancy rate and thus assist in determining whether a patient with elevated P4 level II on hCG day can still receive a fresh ET. In summary, we suggest that a combination of P4 level I (0.975 ng/ml) and the P4 ratio (1.62) maybe more accurate than other parameters in predicting implantation and live birth in normal responders with P4 level II >1.5 ng/ml.

Our results are consistent with those of Melo et al. (14), who reported that serum P4 elevation changes the implantation window more than it affects embryo quality. In this study, the day 3 good embryo rates were similar among all groups; therefore, we suggest that early P4 level I elevation may not affect embryo quality. Consequently, despite transfer of a sufficient number of high-quality embryos, serum P4 level I might lead to endometrium–embryo asynchrony and implantation failure. In addition, the P4 level I > 0.975 ng/ml group in this study demonstrated a significantly higher E2 level on hCG day than that observed in P4 level I ≤ 0.975 ng/ml patients with elevated P4. A higher P4 elevation appears to be associated with higher serum E2 levels on hCG day (1, 5). High levels of E2 and P4 during ovarian stimulation induce secretory transformation of the endometrium (35). Exposure to high E2 concentrations during ovary stimulation may cause endometrial advancement in the late follicular phase by mediating earlier P4 receptor expression (36). Cumulative pregnancy rate has been shown to significantly decrease in extreme endometrial advancement (37, 38). Fluctuations in serum E2 or P4 levels could lead to asynchrony between the embryo and endometrium as well as implantation failure (38, 39). We suggest that the elevated P4 combined with high E2 levels in this study may be related to impaction of implantation in normal responders.

A recent report indicated that P4 elevation in the late follicular phase is related to FSH induced LH receptor expression in granulosa cells (40). The amount of rFSH use may be related to P4 elevation, especially for hypo-responders (40). The report included hypo-, normal and hyper- responders in ART treatment of a randomized clinical trial to evaluate the effect of hCG vs. GnRH agonist trigger for final oocyte maturation (40). However, each follicle within the growth cohort did not contribute the same amount of P4. Only those follicles with granulosa cells rich of LH receptor (mature follicle) would produce a significant level of P4. If the patients with less follicles are in a tendency to have higher average P4 levels, then higher follicular P4 levels will be observed for patients with low anti-Mullerain hormone (AMH) or small number of follicles. However, we have reported that serum AMH levels are associated with follicular FSH levels and the implantation potential of corresponding embryos (41). In that report, the serum AMH is not correlated with follicular P4 levels (41). The mechanism of elevated P4 with poor pregnancy outcome needs further investigation.

A freeze-all policy or blastocyst transfer may appear to be a more suitable solution for women with elevated P4 level II (26). However, the success of these options depends on the patients' embryo quality and the laboratory equipment at the ART center. In this study, fresh ET offered a satisfactory clinical outcome in the subgroup with a lower P4 level I (≤0.975 ng/ml) and higher P4 ratio (>1.62). Our data indicate that given the deleterious effect of elevated P4 on implantation in patients with P4 level I > 0.975 ng/ml, frozen ET may be a better choice. Use of P4 level I and the P4 ratio enables screening of groups that absolutely affect pregnancy rate to determine who should receive frozen ET instead of fresh ET.

One limitation of this study is the retrospective design; therefore, it is not known whether P4 level I and the P4 ratio can serve as indicators prospectively. According to the data in the present study, we recommend the decision tree demonstrated in Figure 2 for the patients with a high P4 on the day of hCG administration. If we took the 39.2% as the live birth rate of the population for fresh ET, then 626, 366, and 700 fresh ET cycles were needed to prove the benefit of a live birth rate at 44.7, 46.4, and 44.4%, respectively. It means we need a larger prospective trial to confirm the benefit of this decision tree models.

Second limitation is that only normal responders were included in this study and the results may thus not be applicable to hyper- or hypo-responders. The elevated P4 levels do affect the pregnancy outcome in fresh ET cycles for normal responders but not hyper-responders (10). Nonetheless, freeze all policy and embryo cumulation are emerging strategy for hyper- and hypo- responders, respectively. The practice of fresh ET for a single oocyte retrieval become rare and rare for hyper- and hypo-responders. Furthermore, to the best of our knowledge, this is the first study to demonstrate the effect of a change in P4 level during the last 2 days of stimulation, rather than of a single level above the defined threshold. Although questions remain regarding the effects of late-follicular-phase P4 elevation, an assessment of change in the last 2 days of the stimulation cycle may be more informative than a single measurement. However, more prospective studies are necessary before any definite conclusions can be drawn.

The third limitation is that the fluctuation of P4 levels between the morning and the evening on the day of hCG trigger (42) may result in the wrong interpretation of elevated P4 levels. In the present study, the blood tests for P4 levels were performed at a range of 9 a.m. to 1 p.m. According to the recent report (42), the gradual increase of P4 levels in these time period might be limited. Furthermore, for every single patient, the time of blood tests within a day is relatively fixed in a range of 2–3 h. Therefore, the ratio of P4 level II/ level I might be consistent under such condition. Although the absolute values of P4 level I or P4 level II might have some bias due to the various time of measurement, the P4 ratio still provided significant value to predict the pregnancy outcome for fresh ET in normal responders.

## Conclusions

In conclusion, available evidence suggests that elevation of circulating P4 level I in the last 2 days before hCG administration has a negative effect on implantation and live birth rates in normal responders. In this study, patients with serum P4 level I ≤ 0.975 ng/ml showed the best predicted clinical outcomes after fresh ET, indicating that these patients may consider choosing to receive fresh ET to reduce the duration of ART treatment and the cost of embryo cryopreservation. By contrast, patients with P4 ratio ≤ 1.62 and P4 level I > 0.975 ng/ml demonstrated the poorest predicted live birth rate with fresh ET; therefore, they may avoid fresh ET. We suggest that freeze all and subsequent FET may be a better choice for this group. This study may assist physicians in establishing criteria for elevated P4 and assessing its possible negative effects on pregnancy and live birth rates in normal responders, thus helping in deciding on whether to recommend fresh ET.

## Data Availability Statement

All datasets generated for this study are included in the Supplementary Material.

## Ethics Statement

The studies involving human participants were reviewed and approved by the Institutional Review Board of Chung Shan Medical University, Taichung, Taiwan. The ethics committee waived the requirement of written informed consent for participation.

## Author Contributions

C-IL, M-SL, and T-HL contributed to the conception and design. C-IL, H-HC, P-YL, and C-CH acquired, analyzed, and interpreted the data. H-HC and T-HL drafted the article. M-SL and T-HL revised the article critically for important intellectual content. All authors have approved the final version of this manuscript.

## Conflict of Interest

The authors declare that the research was conducted in the absence of any commercial or financial relationships that could be construed as a potential conflict of interest.

## Supplementary Material

Supplementary Table 1 P4 data.xlsx The data used in the present study.
